# Illumina sequencing of clinical samples for virus detection in a public health laboratory

**DOI:** 10.1038/s41598-019-41830-w

**Published:** 2019-04-01

**Authors:** Bixing Huang, Amy Jennison, David Whiley, Jamie McMahon, Glen Hewitson, Rikki Graham, Amanda De Jong, David Warrilow

**Affiliations:** 1 Public Health Virology Laboratory, Queensland Health Forensic and Scientific Services, PO Box 594, Archerfield, Queensland 4108 Australia; 2 Public Health Microbiology Laboratory, Queensland Health Forensic and Scientific Services, PO Box 594, Archerfield, Queensland 4108 Australia; 3 Microbiology Division, Pathology Queensland Central Laboratory, Brisbane, Queensland 4029 Australia; 4 Faculty of Medicine, University of Queensland Centre for Clinical Research, The University of Queensland, Brisbane, Queensland 4029 Australia

**Keywords:** Viral infection, Diagnostic markers

## Abstract

High-throughput sequencing (HTS) provides the opportunity, once a diagnostic result is obtained, to extract additional information from a virus-containing sample. Hence, it offers advantages over established quantitative amplification technology, such as quantitative PCR, particularly in a public health environment. At this early stage of its clinical application, there have been limited studies comparing HTS performance to that of the more established quantitative PCR technology for direct detection of viruses. In this pilot-scale study, we tested HTS with a range of viruses and sample types routinely encountered in a public health virology laboratory. In comparison with quantitative PCR, our HTS method was able to sensitively (92%) detect all viruses in any sample type with the exception of certain tissues. Moreover, sufficient nucleotide sequence information was obtained to enable genotyping of strains detected, thus providing additional useful epidemiological information. While HTS sensitivity may not yet match that of PCR, the added value through enhanced epidemiological data has considerable potential to enable real-time surveillance of circulating strains so as to facilitate rapid and appropriate response to outbreaks and virus zoonotic spillover events.

## Introduction

The technology broadly referred to as “next generation sequencing” or high-throughput sequencing (HTS) uses various chemistries to obtain large amounts of data about the nucleic acid composition of a sample. The technology is used in multiple areas from the biological sciences to health. For the latter, it has been applied to the diagnosis of inheritable diseases, cancer, and infectious diseases. HTS potentially enables everything in the sample to be sequenced. This unbiased aspect of HTS has facilitated rapid large-scale virus discovery, as well as the detection of unusual or novel viral agents associated with human disease^1–11^. As well as exploring cases of unknown disease etiology, HTS offers the prospect of combining diagnostics with sequence analyses to reveal clinically relevant information which can be used to enhance public health disease interventions and disease surveillance. In virology, this includes molecular epidemiology and genotyping for outbreak investigations, exclusion testing for other non-viral pathological agents and the identification of antiviral resistance mutations and virulence determinants.

Whilst HTS has become widely adopted in the infectious disease area over the last 10 years, and the technology and supporting bioinformatics substantially improved, it is still considered to be an immature technology. HTS’s strengths complement the polymerase chain reaction (PCR), now considered the primary diagnostic workhorse. However, the frequency with which changes are made to the basic chemistry and the associated hardware and software necessitate ongoing reassessment of its use. Interestingly, whilst there are many examples of its application to microbiological metagenomics and detection of novel agents, there are few examples in the literature comparing its diagnostic efficacy to more conventional assays for direct detection of viruses in clinical samples. Of the studies conducted so far, the most thorough comparisons have involved comparing HTS with PCR for direct detection of viruses in respiratory specimens^12–16^. In these studies, correlations were found between viral load or quantitative PCR cycle threshold number (C_T_; the cycle where the fluorescent signal from the product amplification starts to exceed a baseline value and being a value that is inversely proportional to viral load), and numbers of normalized reads matching the target. Moreover, all the studies except one^16^ observed levels of HTS sensitivity equivalent to, or approximating, that of PCR. Another study investigating hand, foot and mouth disease virus detection in clinical specimens over a range of viral loads also observed comparable sensitivity to reverse–transcription quantitative PCR (RT-qPCR)^17^. A further study using samples spiked with various different viral agents confirmed the importance of viral load, with virus detected by HTS in all samples except those with PCR C_T_ values more than or equal to 37 cycles^18^. Overall HTS technology is looking promising for direct viral detection, however given the limited number of these studies to date, there is a need to examine the technology over a wider range of viruses and sample types.

We are a routine public health virology laboratory, and in this study we sought to explore the utility of HTS methodology in our setting. Briefly, the ability of HTS to directly detect viruses was compared with established PCR assays for viruses and sample types commonly encountered in the viral diagnostic laboratory. The viruses tested included those with single-stranded RNA, double-stranded RNA and double-stranded DNA genomes; and genome sizes from ~10 Kb to ~200 Kb. The clinical samples types tested included serum, urine, feces, swabs of various sites, and brain tissue. In addition to measuring clinical sensitivity, a key aim was to examine the additional nucleotide sequencing data that could be obtained for downstream phylogenetic analysis, so as to assist with outbreak investigations. Molecular epidemiology is an integral part of our public health investigative processes, and so HTS directly on clinical material has the potential to help target effective responses.

## Materials and Methods

### Clinical samples for sequencing

Clinical samples (*n* = 52) of various kinds, including serum (*n* = 18), urine (*n* = 2), feces (*n* = 8), nappy swabs (*n* = 2), skin swabs (*n* = 5), respiratory swabs (*n* = 14), and tissue (*n* = 3), were chosen on the basis of previously being identified as containing a virus by either RT-qPCR (RNA viruses, *n* = 47) or qPCR (DNA viruses, *n* = 5)^19^. These are detailed (virus, sample type, numbers, and C_T_ values) in Table S1 (supplementary file) and comprised a mix of viruses commonly encountered by the laboratory (including influenza, measles, mumps, arboviruses, molluscum contagiosum, rotavirus and noroviruses) and having a spectrum of low to high viral based on previously determined C_T_ values. The samples were submitted to the Public Health Virology Laboratory for routine viral testing over the period January 2015–March 2017. Further details of the PCR assays used for routine diagnostics are provided in Table S2. Please note that sequence data for a chikungunya virus and 7 norovirus samples (indicated in Table S1) were previously published; however, here we further analyzed the data for its diagnostic relevance. All work handing clinical material was done at biocontainment level 3.

### Limit of detection estimation using bovine viral diarrhea virus (BVDV)

A single-stranded and non-segmented RNA virus, an isolate of BVDV (strain MD-73), was used as a source of template for positive control libraries and for limit of detection experiments. The isolate was grown in Madin-Darby bovine kidney cells (MDBK) by serial passage at 37 °C in growth medium (Opti-MEM with 3% fetal calf serum). RNA was extracted from tissue culture fluid as for clinical samples. BVDV genomic RNA was serially diluted and the C_T_ value determined for each dilution by RT-qPCR. Dilutions with C_T_ values of 19, 25, 30, 33 and 37 were used in the construction of Illumina Nextera cDNA libraries as described for clinical samples above.

### High-throughput virus genome sequencing

Nucleic acid was extracted manually using the QIAamp Viral RNA extraction kit (Qiagen) as per the manufacturer’s instructions, omitting carrier RNA from the sample. Depending on whether the virus was RNA or DNA (based on the original PCR results), either an RNA or DNA library was prepared. RNA libraries were prepared as described previously^20^ using an equivalent amount of extract as for qPCR (5 µL of 60 µL total). Briefly, host DNA was removed by DNase treatment using heat-labile Heat & Run (ArcticZymes) followed by inactivation at 80°C for 5 min. First-strand cDNA was generated using Protoscript II kit (New England Biolabs) with the supplied random primer mix followed by second strand DNA synthesis using a cocktail of enzymes including *Escherichia coli* DNA ligase, DNA polymerase I, and RNase H (New England Biolabs). Libraries were constructed with the Nextera XT library kit (Illumina) using barcoded primers. A negative control library prepared from fetal calf serum and a positive control library prepared from a BVDV RNA spiked sample (diluted to C_T_ 25), were also included. DNA libraries were prepared as for RNA libraries, omitting a DNase pre-treatment and cDNA synthesis steps. Library quality was assessed on a TapeStation prior to sequencing. Libraries passing the quality check were sequenced with a v2 mid-output kit on an Illumina NextSeq 500 machine with a minimum of 4.6 million reads (2 × 150 nt paired).

### Genome assembly

Bioinformatics was performed using a method developed in-house. Draft genomes were assembled by mapping reads to closely-related complete reference sequences obtained manually from GenBank (https://www.ncbi.nlm.nih.gov/) using the inbuilt Geneious Mapper at the lowest sensitivity settings with no fine tuning on Geneious R11 software^21^. The proportion of reads corresponding to the viral target was determined by calculating the normalized reads (matches per million total reads, per kilobase total genome size). Genome sizes were obtained from ViralZone https://viralzone.expasy.org/. Normalized reads were presented as boxplots, and were constructed using the online version of BoxPlotR (http://shiny.chemgrid.org/boxplotr/).

### Phylogenetic analysis

Molluscum contagiosum genome alignments were performed with Mauve^22^ as it can process genomes on this scale (~200 Kb). Measles and mumps virus genome alignments were performed with MAFFT v7.388^23^ (algorithm set to “Auto”; scoring matrix 200PAM/k = 2; a gap open penalty of 1.53; and an offset value of 0.123). Phylogenetic trees were constructed from the alignments with FastTree v2.1.5^24^ as part of the Geneious R11 package (Jukes-Cantor model, 20 categories of sites and optimized for Gamma20). Branch support values were calculated using the Shimodaira-Hasegawa test and are shown as a percentage.

### Ethical approval

This work was approved by the Queensland Health Forensic and Scientific Services Human Ethics Committee in accordance with the Australian NHMRC National Statement on Ethical Conduct in Human Research 2007. The committee decided that the need for patient consent was waived for this study. All samples were submitted for routine diagnostics and had been de-identified. It should be noted that a condition of the ethical approval was that we were restricted to detecting only viruses known to be present in the sample and were prevented from attempting to use HTS to detect viruses other than those specified in the original pathology request. Hence, we were unable to determine the specificity of the HTS method.

## Results

### Limit of detection using BVDV

**Figure 1:**
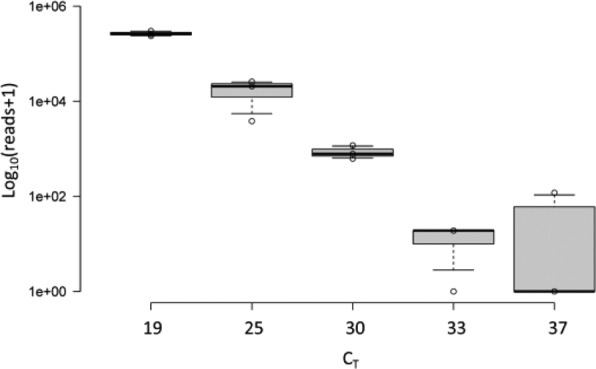
An estimation of the limit of detection for BVDV detection. (**A**) logarithmic representation of the matching reads in relation to the C_T_ value is shown for each dilution of BVDV. Individual data points are given (°); the boxplots give the 1^st^ and 3^rd^ quartile, and whiskers 5^th^ and 95^th^ percentile.

BVDV RNA was used as a surrogate to enable an estimation of the approximate limit of detection of HTS for clinical samples. Libraries were constructed in triplicate from samples spiked with serially diluted BVDV. A comparison of the C_T_ values with the logarithm of the normalized reads matching the target (Fig. 1) revealed a correlation (*r* = −0.97, Pearson’s correlation co-efficient, *P* < 0.05; *R*^2^ = 0.95). HTS detection was reproducible except for at the lowest BVDV RNA concentrations where no reads were detected in some replicates; virus was detected by HTS in two of three replicates at a C_T_ of 33 and only one of three replicates at a C_T_ of 37. Our accepted cut-off for detection is normally a C_T_ of <40. Assuming product doubling every cycle, these data therefore show that the reliable limit of detection of HTS is approximately 1–2 logs less than that of qPCR; albeit, the C_T_ being only an approximation, not an absolute measure, of viral load.

### Detection of virus in clinical samples using HTS

**Figure 2:**
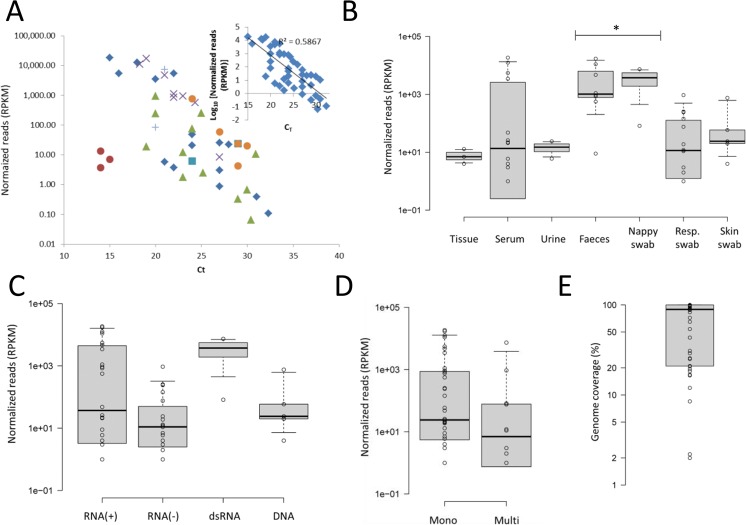
HTS assay performance with virus genomes and sample types. (**A**) Normalized reads (matching reads per million total reads per genome size, RPKM) are given for each sample type (tissue, ; serum, ; urine, ; feces, ; nappy swab, ; respiratory swab, ; and skin swab, ). Data was used to calculate a correlation (inset). (**B**) Sample types data represented as boxplots. Samples that were significantly different to the other types are indicated (^*^P < 0.01; Mann-Whitney). Normalized reads comparing (**C**) genome nucleic acid and (**D**) partite structure. (**E**) Coverage obtained shown as a percentage of total genome size. Individual data points are shown (_°_); the boxplots give the 1^st^ and 3^rd^ quartile, and whiskers 5^th^ and 95^th^ percentile.

Libraries were prepared from a selection of 52 specimens shown to contain virus by qPCR. HTS detected the respective virus in 48 (92%) of these samples. With the exception of brain tissue (*n* = 3), reads matching the target and normalized to genome size (Fig. 2A) were inversely correlated with the C_T_ value (*r* = -0.76, Pearson’s correlation co-efficient, *P* < 0.05; *R*^2^ = 0.59; Note that samples with no matches to the target were excluded from calculation of the co-efficient). In the case of brain tissue, the number of matching reads was lower than that expected from the C_T_ value. It is likely that residual DNA from the sample competed with the viral target cDNA for incorporation into the library, and suggests the method may need further optimization for sequencing tissue.

A boxplot of normalized reads for each sample type is provided in Fig. 2B. Fecal samples (feces and nappy swab) had higher median values than other sample types (*P* < 0.01, Mann-Whitney test). This indicated higher viral loads in these samples, and was most likely due the fact that they primarily contained norovirus, which is known to have a high viral load in feces. With regard to genome type, only the dsRNA had noticeably higher normalized reads but was not statistically significant (Fig. 2C). There was no significant difference between RNA and DNA genomes (*P* = 0.84, Mann-Whitney test), plus-sense ssRNA and minus-sense ssRNA (*P* = 0.10, Mann-Whitney test); however, this was on the basis of 5 DNA virus samples and ideally more should be tested. Nor was there a significant difference between monopartite and multipartite genomes (Fig. 2D), both RNA and DNA included (*P* = 0.12, Mann-Whitney test). Hence, with the exception perhaps of tissue, the method is amenable to all sample and viral genome types.

**Figure 3:**
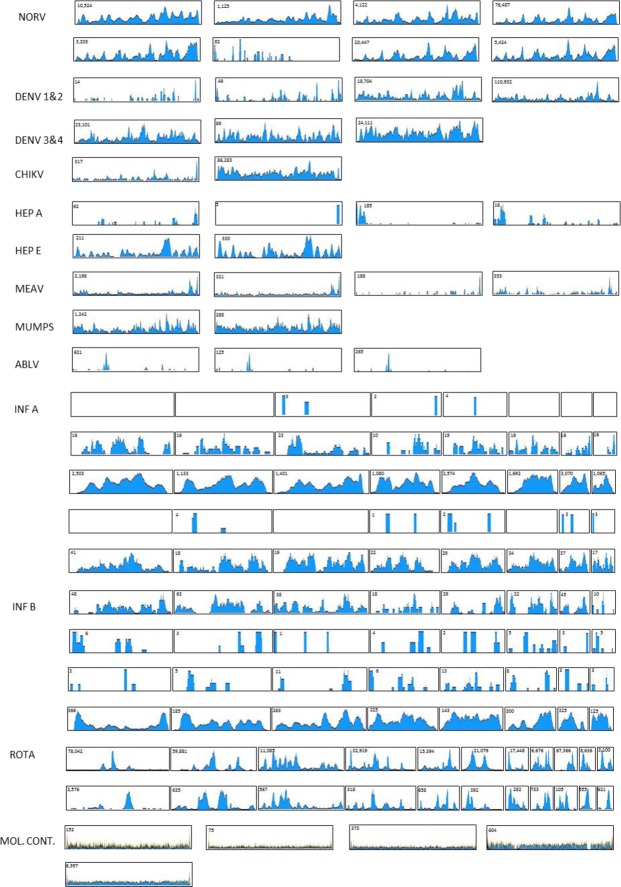
HTS contiguous assemblies. Assemblies are shown for each of the positive samples. Viruses shown are norovirus (NORV), dengue viruses 1 and 2 (DEN 1&2), dengue viruses 3 and 4 (DEN 3&4), chikungunya virus, (CHIKV), hepatitis A (HEP A), hepatitis E (HEP E), measles virus (MEAV), mumps virus (MUMPS), Australian bat lyssavirus (ABLV), influenza A virus (INF A), influenza B virus (INF B), rotavirus (ROTA), molluscum contagiosum (MOL. CONT.). Each box presents a single contiguous sequence (contig). Multi-partite genome assemblies are shown in size relative to their other genome segments. Other contigs are all presented as the same size regardless of the genome length. The maximum read depth for each contig is given in the top left-hand corner of each assembly diagram.

Genome coverage was defined as the percentage of genome for which a contiguous sequence was obtained. The median genome coverage (Fig. 2E) for all viruses was 89% (20, 100; 1^st^ and 3^rd^ quartiles, respectively). Genome coverage for all viruses is shown graphically (Fig. 3). Interestingly, where there were sufficient reads obtained, each virus species seemed to have a distinctive and remarkably consistent read profile. This suggests one, or a combination, of two possibilities. Firstly, primer binding for reverse transcription is dependent on the structure of the RNA genome. This explanation is supported by the seemingly flatter read profile of the molluscum contagiosum samples; however, the much larger DNA genome size of this virus could make this deceptive. The uneven profile of the rotavirus samples with dsRNA genome, which should have minimal genome structure, argues against this. Alternatively, aspects of the library creation process, such as the transposase recombination step, may not be entirely random, leading to biases in those sequences incorporated into the library.

### Genotyping for disease surveillance and outbreak investigations

**Figure 4:**
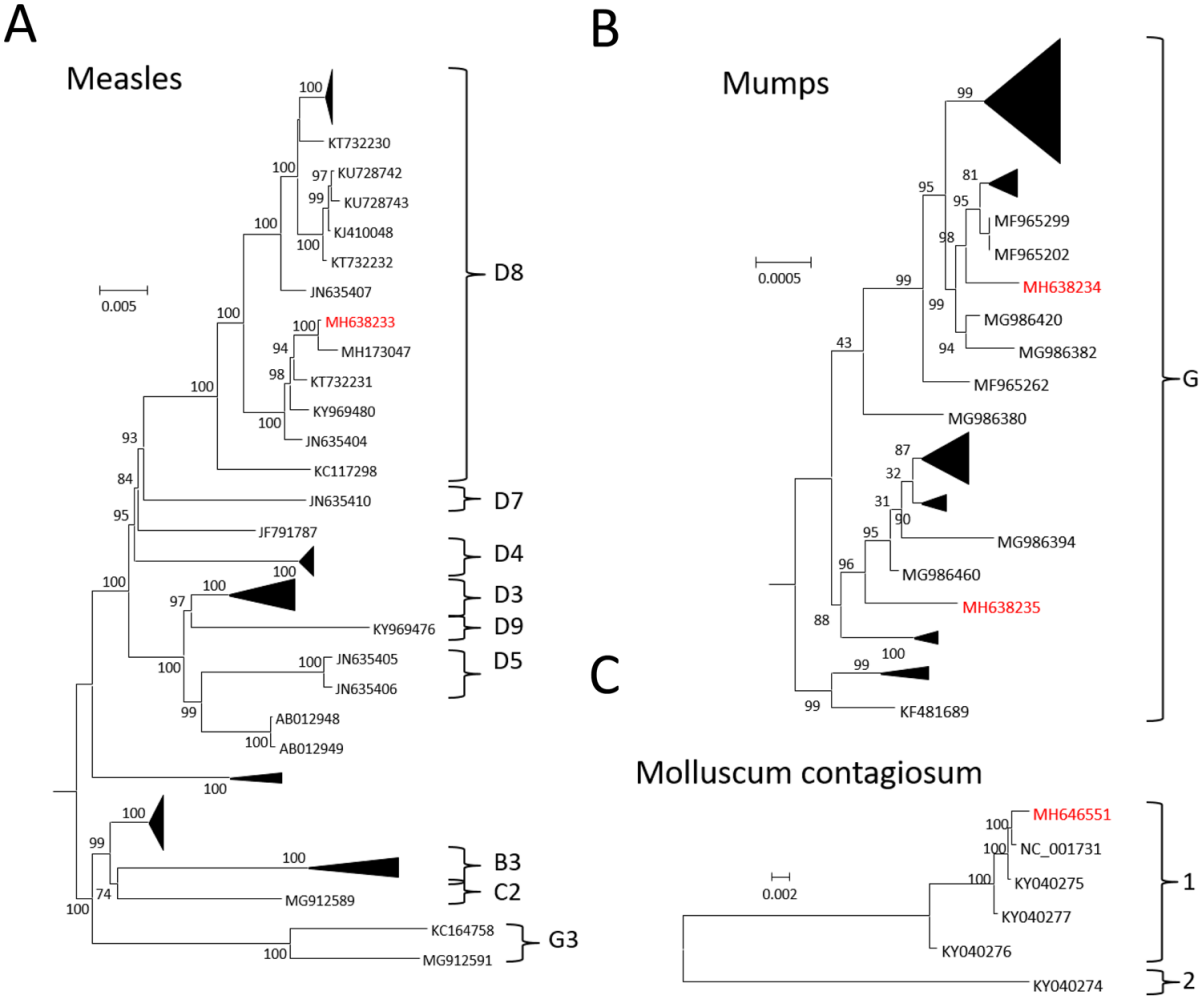
Virus genotyping using phylogenetic methods. Genome alignments were used to generate phylogenetic trees using FastTree for (**A**) measles, (**B**) mumps and (**C**) molluscum contagiosum viruses. The virus sequences originating in this study are shown as their GenBank accession numbers and are depicted in red. The other viruses are represented as their corresponding accession numbers. Genotypes are given where this information was provided. Branch support is given as a percentage.

The epidemiology of some samples included in this study, a case of importation of chikungunya virus and 7 noroviruses cases associated with outbreaks in aged care facilities, were published previously^20,25^. Phylogenetic-based genotyping was attempted for an additional 4 of the 48 samples providing genome sequence data, and these samples are indicated in Table S1. A nucleotide alignment was performed on the measles genome sequence obtained from a 21 year-old female. Branch support confidently placed the genome in the genotype D8 (Fig. 4A). This was consistent with previous observations that the D8 genotype was the second highest prevalent genotype responsible for cases reported in New South Wales, Australia, over the period 2002–2011^26^. Identical methodology was used to type two cases of mumps virus infection. One sample was from a 32 year-old female and the other from a 49 year-old male. The latter showed classic parotitis on the left side. These were both placed within the G genotype (Fig. 4B), but from different circulating strains due to the genetic distance separating the two sequences. We are not aware of this genotype being reported in Australia before; however, it has been widely reported in Western countries in recent years^27^. Finally, a molluscum contagiosum genome from a 63 year-old male (Fig. 4C) was able to be genotyped as subtype 1, the most prevalent subtype^28^. These examples, and those from the previously published cases, demonstrate the value of application of this technology to public health.

## Discussion

There is a clear need to determine how unbiased HTS performs with different viruses and sample types. Here, we conducted a pilot study using the most commonly used HTS platform (Illumina). In this setting, the detection of associated unusual and novel viral agents is a real possibility. Hence, we have used minimal sample and data manipulation (i.e. no filtration) to reduce the possibility of bias. Our main interest is the direct detection of RNA viruses frequently encountered by our public health laboratory. In doing so, we examined HTS capability to detect viruses of various genome sizes and types, and in a variety of commonly encountered clinical samples and viral loads. This is a major potential advantage of using HTS over conventional methods as a diagnostic tool. In comparison with qPCR, the method was found to detect 92% of the positive samples. Our results compare favorably with the other similar studies described previously^12–16^, and extends the comparison of HTS with PCR to clinical material other than previously published studies on respiratory samples.

Similarly to the previous comparative studies, HTS normalized reads from the various samples (tissue excepted) were inversely correlated to C_T_ values, and were found to approximate the line of best fit. This suggested that the amount of non-viral nucleic acids competing for library incorporation in these samples was similar. This observation was surprising, as it was expected that different sample types might vary in the relative amount of nucleic acid from the host and contaminating bacteria. It suggested that any of these samples may be used for HTS provided that viral load is high enough, and the number of sequencing reads is sufficient. Regarding the latter, the result also indicates that the minimum number of reads to be collected to be confident of virus detection could be similar for most sample types. We used approximately 5 million reads as a minimum, however further assessments of this may be warranted given the relatively low number of individual sample types tested.

Importantly, from a public health investigation perspective, we were able to use HTS to identify both previously identified and unidentified genotypes directly in clinical samples. This included information on an imported case of chikungunya virus infection; and genotype information of strains related to norovirus outbreaks in aged care facilities, measles, mumps and molluscum contagiosum. Notably, this ability to obtain significant epidemiologically useful sequence information directly from clinical samples represents a key potential use of HTS to public health laboratories with access to this technology. That is, even if HTS cannot ultimately match the diagnostic sensitivity of PCR, it could otherwise prove to be an invaluable reflex test to PCR where targeted PCR methodology has been unable to identify a causative agent, for surveillance purposes, and to provide enhanced epidemiological information during an outbreak. Certainly, our intention is not to suggest that HTS is a replacement for PCR.

Ethical restrictions meant we were unable to test for viruses other than those originally requested, making a false positive determination and assessment of specificity impossible. However, it is expected that with the use of stringent assembly and blast search criteria, detection should be highly specific. Nevertheless, the fact that ethics committee did raise such initial concerns about use of the methodology highlights a potential limitation of regarding future use. For example, will clinicians be keen to adopt this given the potential for incidental findings? In our opinion this will hinge on the importance of the clinical question, whereby the risks posed by ancillary findings are outweighed by the need to accurately diagnose and treat the patient.

As the study was restricted to ~50 samples, there were unavoidable biases introduced with a study of this size, such as different frequencies and species of virus genomes found in different sample types. Due to the relatively small number of DNA virus samples tested, further work needs to be done with this group of viruses to be confident of detection. Hence, there is still a need to expand this work to a much larger study to ensure widespread applicability of the method which includes further optimization with regard to all kinds of samples and viral genome types. Additionally, some aspects of the HTS method could be improved. For example, due the ethical restrictions discussed above, draft genome assembly was limited to reference assembly only. Preferably, a *de novo* assembly such as SPAdes^29^ should be used to minimize the level of bias and detect additional agents if these were present.

Recent work has highlighted the importance of genome data for identifying and responding to virus emergence in which co-circulating viruses can cause clinically similar manifestations among human hosts^30,31^. Genomic data is of great value in the early stages of emergence (i.e. spillover events) when the nature of a novel agent is unknown^32^, to determine movement and spread during the outbreak, and changes to the genome which might affect the ability to detect the virus or develop effective vaccines or other therapeutics. Targeted real-time surveillance of vulnerable populations has been identified as one of the most effective measures to flag when more dramatic public health interventions are required. We believe that widespread application of virus genome sequencing to routine virus diagnostics will greatly facilitate this real-time surveillance strategy. To make this a reality, a number of previously identified challenges will need to be overcome^33^. In our hands, RNA libraries were constructed for ~$US120 including labour, and DNA libraries for ~$20 less. Turn-around time was 48 h for sample preparation and library construction, and 29 h for Illumina sequencing. These will need to be reduced, and further consideration given to improving sensitivity, reducing contamination, dealing with ethical and quality control and assurance concerns, and appropriate bioinformatic support for ease of clinical interpretation. Additional studies are needed to fully examine the actual utility of such methods in epidemiological investigations during outbreak response. As a first step, this work demonstrates that broader application of HTS in clinical virology is feasible, and gives examples of its application to sequencing of clinical samples directly to enable epidemiological information to be extracted.

## Supplementary information

Supplementary information

## Data Availability

All data generated or analyzed during this study are included in this published article (and its Supplementary Information files).

## References

[1] Brown JR, Bharucha T, Breuer J (2018). Encephalitis diagnosis using metagenomics: application of next generation sequencing for undiagnosed cases. J Infect.

[2] Chan BK, Wilson T, Fischer KF, Kriesel JD (2014). Deep sequencing to identify the causes of viral encephalitis. PLoS One.

[3] Chiu CY (2013). Viral pathogen discovery. Curr Opin Microbiol.

[4] Chiu CY (2017). Diagnosis of Fatal Human Case of St. Louis Encephalitis Virus Infection by Metagenomic Sequencing, California, 2016. Emerg Infect Dis.

[5] Chiu CY (2008). Identification of cardioviruses related to Theiler’s murine encephalomyelitis virus in human infections. Proc Natl Acad Sci USA.

[6] Fremond ML (2015). Next-Generation Sequencing for Diagnosis and Tailored Therapy: A Case Report of Astrovirus-Associated Progressive Encephalitis. J Pediatric Infect Dis Soc.

[7] Lysholm Fredrik, Wetterbom Anna, Lindau Cecilia, Darban Hamid, Bjerkner Annelie, Fahlander Kristina, Lindberg A. Michael, Persson Bengt, Allander Tobias, Andersson Björn (2012). Characterization of the Viral Microbiome in Patients with Severe Lower Respiratory Tract Infections, Using Metagenomic Sequencing. PLoS ONE.

[8] Naccache SN (2015). Diagnosis of neuroinvasive astrovirus infection in an immunocompromised adult with encephalitis by unbiased next-generation sequencing. Clin Infect Dis.

[9] Smits SL (2012). Calicivirus from novel Recovirus genogroup in human diarrhea, Bangladesh. Emerg Infect Dis.

[10] Stremlau MH (2015). Discovery of novel rhabdoviruses in the blood of healthy individuals from West Africa. PLoS Negl Trop Dis.

[11] Yozwiak Nathan L., Skewes-Cox Peter, Stenglein Mark D., Balmaseda Angel, Harris Eva, DeRisi Joseph L. (2012). Virus Identification in Unknown Tropical Febrile Illness Cases Using Deep Sequencing. PLoS Neglected Tropical Diseases.

[12] Fischer N (2015). Evaluation of Unbiased Next-Generation Sequencing of RNA (RNA-seq) as a Diagnostic Method in Influenza Virus-Positive Respiratory Samples. J Clin Microbiol.

[13] Graf EH (2016). Unbiased Detection of Respiratory Viruses by Use of RNA Sequencing-Based Metagenomics: a Systematic Comparison to a Commercial PCR Panel. J Clin Microbiol.

[14] Greninger AL (2010). A metagenomic analysis of pandemic influenza A (2009 H1N1) infection in patients from North America. PLoS One.

[15] Prachayangprecha S (2014). Exploring the potential of next-generation sequencing in detection of respiratory viruses. J Clin Microbiol.

[16] Thorburn F (2015). The use of next generation sequencing in the diagnosis and typing of respiratory infections. J Clin Virol.

[17] Nguyen AT (2016). Development and evaluation of a non-ribosomal random PCR and next-generation sequencing based assay for detection and sequencing of hand, foot and mouth disease pathogens. Virol J.

[18] Li L (2015). Comparing viral metagenomics methods using a highly multiplexed human viral pathogens reagent. J Virol Methods.

[19] van den Hurk AF, Hall-Mendelin S, Pyke AT, Smith GA, Mackenzie JS (2010). Vector competence of Australian mosquitoes for chikungunya virus. Vector Borne Zoonotic Dis.

[20] Huang, B., Pyke, A. T., McMahon, J. & Warrilow, D. Complete Coding Sequence of a Case of Chikungunya Virus Imported into Australia. *Genome Announc* **5**, 10.1128/genomeA.00310-17 (2017). 10.1128/genomeA.00310-17 PMC5427210 28495775

[21] Kearse M (2012). Geneious Basic: An integrated and extendable desktop software platform for the organization and analysis of sequence data. Bioinformatics.

[22] Darling AC, Mau B, Blattner FR, Perna NT (2004). Mauve: multiple alignment of conserved genomic sequence with rearrangements. Genome Res.

[23] Katoh K, Standley DM (2013). MAFFT multiple sequence alignment software version 7: improvements in performance and usability. Mol Biol Evol.

[24] Price MN, Dehal PS, Arkin AP (2010). FastTree 2–approximately maximum-likelihood trees for large alignments. PLoS One.

[25] Lun JH (2018). Emerging recombinant noroviruses identified by clinical and waste water screening. Emerg Microbes Infect.

[26] Rosewell A, Reinten-Reynolds T, Spokes PJ (2012). EpiReview: Measles in NSW, 2002-2011. N S W Public Health Bull.

[27] Sabbe M, Vandermeulen C (2016). The resurgence of mumps and pertussis. Hum Vaccin Immunother.

[28] Lopez-Bueno A, Parras-Molto M, Lopez-Barrantes O, Belda S, Alejo A (2017). Recombination events and variability among full-length genomes of co-circulating molluscum contagiosum virus subtypes 1 and 2. J Gen Virol.

[29] Bankevich A (2012). SPAdes: a new genome assembly algorithm and its applications to single-cell sequencing. J Comput Biol.

[30] Grubaugh ND, Faria NR, Andersen KG, Pybus OG (2018). Genomic Insights into Zika Virus Emergence and Spread. Cell.

[31] Holmes EC, Rambaut A, Andersen KG (2018). Pandemics: spend on surveillance, not prediction. Nature.

[32] Woolhouse ME, Brierley L, McCaffery C, Lycett S (2016). Assessing the Epidemic Potential of RNA and DNA Viruses. Emerg Infect Dis.

[33] Allcock RJN, Jennison AV, Warrilow D (2017). Towards a Universal Molecular Microbiological Test. J Clin Microbiol.

