# Author Correction: Illumina sequencing of clinical samples for virus detection in a public health laboratory

**DOI:** 10.1038/s41598-019-50827-4

**Published:** 2019-10-18

**Authors:** Bixing Huang, Amy Jennison, David Whiley, Jamie McMahon, Glen Hewitson, Rikki Graham, Amanda De Jong, David Warrilow

**Affiliations:** 1 Public Health Virology Laboratory, Queensland Health Forensic and Scientific Services, PO Box 594, Archerfield, Queensland 4108 Australia; 2 Public Health Microbiology Laboratory, Queensland Health Forensic and Scientific Services, PO Box 594, Archerfield, Queensland 4108 Australia; 3 Microbiology Division, Pathology Queensland Central Laboratory, Brisbane, Queensland 4029 Australia; 4 Faculty of Medicine, University of Queensland Centre for Clinical Research, The University of Queensland, Brisbane, Queensland 4029 Australia

Correction to: *Scientific Reports* 10.1038/s41598-019-41830-w, published online 01 April 2019

The original version of this Article contained a typographical error in the spelling of the author Amy Jennison, which was incorrectly given as Amy Jennsion.

Additionally, the original version of this Article contained a typographical error in the Materials and Methods section under the subheading ‘Limit of detection estimation using bovine viral diarrhea virus (BVDV)’ where,

“The isolate was grown in Madin-Darby bovine kidney cells (MDBK) by serial passage at 37 °C in growth medium (Optiprep with 3% fetal calf serum).”

now reads:

“The isolate was grown in Madin-Darby bovine kidney cells (MDBK) by serial passage at 37 °C in growth medium (Opti-MEM with 3% fetal calf serum).”

These errors have now been corrected in the PDF and HTML versions of the Article, and in the accompanying Supplementary Information file.

