# Supplementary material for: Illumina sequencing of clinical samples for virus detection in a public health laboratory
Source: Sci Rep. 2019 Apr 1;9:5409. doi: 10.1038/s41598-019-41830-w (PMC6443674; doi:10.1038/s41598-019-41830-w)
Supplement: Supplementary file 1 — Supplementary information [file 41598_2019_41830_MOESM1_ESM.pdf]

Supplementary information

Illumina sequencing of clinical samples for virus detection in a public health laboratory

Bixing Huang[1], Amy Jennison[2], David Whiley[3], Jamie McMahon[1], Glen Hewitson[1], Rikki Graham[2], Amanda De Jong[1], and David Warrilow[1*]

[1]Public Health Virology Laboratory, Queensland Health Forensic and Scientific Services, PO Box 594, Archerfield, Queensland 4108, Australia.

[2]Public Health Microbiology Laboratory, Queensland Health Forensic and Scientific Services, PO Box 594, Archerfield, Queensland 4108, Australia.

[3]Microbiology Division, Pathology Queensland Central Laboratory, Brisbane, Queensland 4029, Australia.

[4]Faculty of Medicine, University of Queensland Centre for Clinical Research, The University of Queensland, Brisbane, Queensland 4029, Australia;

[*]Corresponding author

David Warrilow

Email: David.Warrilow@health.qld.gov.au

Phone: 61-7-30962898

Fax: 61-7-30962878

Table S1. Sample quantitative PCR data and HTS read numbers

Virus	Sample type	C_T	Total reads	Matches per million reads	Genome coverage (%)	Genome size (Kb)[1]	Normalized reads[2]	Accession number
**Australian bat lyssavirus**	Tissue (brain)	14	9137984	159	16.8	11.82	13.46	-
**Australian bat lyssavirus**	Tissue (brain)	14	6319444	45	12	11.82	3.78	-
**Australian bat lyssavirus**	Tissue (brain)	15	7593814	83	16.5	11.82	6.99	-
**Chikungunya**	Serum	24	11164658	247	83	11.83	20.88	-
**Chikungunya**	Serum	34	10339674	0	0	11.83	0.00	-
**Chikungunya**[1]	Serum	15	12318738	219931	100	11.83	18590.93	-
**Dengue 1**	Serum	30	19559010	4	29.1	10.74	0.39	-
**Dengue 1**	Serum	21	6793582	62	43.4	10.74	5.81	-
**Dengue 2**	Serum	15	10524243	37181	98.4	10.74	3461.89	-
**Dengue 2**	Serum	18	11719336	134420	99.7	10.74	12515.81	-
**Dengue 3**	Serum	16	10239496	58868	99.5	10.74	5481.17	-
**Dengue 4**	Serum	25	8839152	240	98.6	10.74	22.30	-
**Dengue 4**	Serum	19	14528866	58991	100	10.74	5492.64	-
**Hepatitis A**	Serum	32	6096794	1	2	7.48	0.11	-
**Hepatitis A**	Serum	22	20579260	28	18.4	7.48	3.76	-
**Hepatitis A**	Serum	27	10933708	6	30.9	7.48	0.87	-
**Hepatitis A**	Serum	27	10408634	23	19.4	7.48	3.06	-
**Hepatitis E**	Serum	27	10712770	183	88	7.18	25.53	-
**Hepatitis E**	Serum	40	4779048	0	0	7.18	0.00	-
**Hepatitis E**	Serum	24	11830270	344	89.3	7.18	47.94	-
**Influenza A**	Resp. swab	20	9887420	12873	100	13.65	943.10	-
**Influenza A**	Resp. swab	29	6362958	5	8.5	13.65	0.33	-
**Influenza A**	Resp. swab	23	7797928	165	92.1	13.65	12.08	-
**Influenza A**	Resp. swab	30	12274192	1	2.2	13.65	0.07	-

**Influenza A**	Resp. swab	25	15933624	34	65	13.65	2.51	-
**Influenza B**	Resp. swab	37	10522006	0	0	14.45	0.00	-
**Influenza B**	Resp. swab	31	8803350	154	72	14.45	10.63	-
**Influenza B**	Resp. swab	30	9078836	10	20.8	14.45	0.68	-
**Influenza B**	Resp. swab	23	5493902	25	25.6	14.45	1.76	-
**Influenza B**	Resp. swab	24	12186300	1093	89	14.45	75.61	-
**Measles**	Resp. swab	20	13806054	3839	99.7	15.89	241.62	MH638233
**Measles**	Urine	24	8575252	100	21	15.89	6.27	-
**Measles**	Urine	29	9648054	382	54	15.89	24.07	-
**Measles**	Resp. swab	19	13776058	299	97.2	15.89	18.79	-
**Molluscum**	Skin swab	30	11068638	3777	99.7	190.29	19.85	-
**Molluscum**	Skin swab	29	11429878	814	94	190.29	4.28	-
**Molluscum**	Skin swab	29	13520942	4536	99.6	190.29	23.84	-
**Molluscum**	Skin swab	27	11739384	11197	88.7	190.29	58.84	-
**Molluscum**	Skin swab	24	15917974	144039	99.9	190.29	756.94	MH646551
**Mumps**	Resp. swab	25	11330012	3913	100	15.38	254.43	MH638234
**Mumps**	Resp. swab	26	5385648	2219	100	15.38	144.28	MH638235
**Norovirus G2[4]**	Feces	18	2928082	84897	100	7.65	11097.67	-
**Norovirus G2[4]**	Feces	24	4136760	4296	100	7.65	561.58	-

**Norovirus G2**[4]	Feces	23	11709848	7149	100	7.65	934.52	-
**Norovirus G2**[4]	Feces	19	10058556	130998	100	7.65	17123.95	-
**Norovirus G2**[4]	Feces	22	10054576	6488	100	7.65	848.06	-
**Norovirus G2**	Feces	27	9946020	65	25.1	7.65	8.50	-
**Norovirus G2**[4]	Feces	21	9630208	36161	100	7.65	4726.92	-
**Norovirus G2**[4]	Feces	22	11135966	8470	100	7.65	1107.19	-
**Rotavirus**	Nappy swab	21	9006280	137170	100	18.56	7390.63	-
**Rotavirus**	Nappy swab	20	6890806	1531	99.6	18.56	82.49	-
**Zika**	Serum	38	11849692	0	0	10.79	0.00	-

1. From ViralZone (https://viralzone.expasy.org/).
2. Matching reads per million reads per kilobase of genome.
3. Genome sequence previously published [1].
4. Genome sequences previously published [2].

Table S2. Primers and probes used in quantitative PCR assays

Virus	Primer or probe	Sequence	Reference
**Bovine viral diarrhea virus**	BVDV-F	TAGCCATGCCCTTAGTAGGAC	[3]
	BVDV-R	GACGACTACCCTGTACTCAGG	"
	BVHD1-FAM	FAM-AACAGTGGTGAGTTCGTTGGATGGCTT-TAMRA	"
**Measles**	Measles MGB FP	GCTCAAATTGCTCAGATACTATACAGAAA	[4]
	Measles MGB RP	GCAGATATGGGGTCCCGTAA	"
	Measles MGB Probe	FAM-CCTGTCATTATTTGGCC-MGBNFQ	"
**Chikungunya**	CHIK-MAfor	CCCGGTAAGAGCGGTGAA	[5]
	CHIK-MArev	CTTCCGGTATGTCGATGGAGAT	"
	CHIK-MAprobe	FAM-TGCGCCGTAGGGAACATGCC-TAMRA	"
**Hepatitis A**	Hep A FP	GCTCTGGCCGTTGCG	[6]
	Hep A RP	CCCCAATTTAGACTCCTACAGCTC	"
	Hep A MGB probe	FAM-TCATGGAGTTGACCCCGCC-MGBNFQ	"

**Hepatitis E**	JVHEVF	GGTGGTTTCTGGGGTGAC	7
	JVHEVR	AGGGGTTGGTTGGATGAA	"
	JVHEVPmod MGB	FAM-TGATTCTCAGCCCTTCGC-MGBNFQ	"
**Norovirus G2**	NORO G1 TAQ F COG1F	CGYTGGATGCGNTTYCATGA	8
	NORO G1 TAQ R COG1R	CTTAGACGCCATCATCATTYAC	"
	NORO G1 TAQ PROBE RING1(a)-TP	VIC-AGATYGCGATCYCCTGTCCA-TAMRA	"
	NORO G1 TAQ PROBE RING1(b)-TP	VIC-AGATCGCGGTCTCCTGTCCA-TAMRA	"
	NORO GII TAQ F COG2F	CARGARBCNATGTTYAGRTGGATGAG	"
	NORO GII TAQ R COG2R	TCGACGCCATCTTCATTCACA	"
	NORO GII TAQ PROBE RING2-TP	FAM-TGGGAGGGCGATCGCAATCT-TAMRA	"
**Australian bat lyssavirus**	LYS INT FFF	GGAATGAATGCTGCAAAGCTG	9
	LYS EXT R	GGCAGAYCCCCTCAAATAACTC	"
	LYS FF - FAM	FAM-ACCCCGATGATGTATGTTCTTACTTAGCTGCAG-TAMRA	"
**Influenza A (H1N1)**	SwFluH1fwd	CCCCATTGCATTTGGGTAAA	10
	SwFluH1rev	TGGAGAGTGATTCACACTCTGGAT	"
	SwFluH1Prb	FAM-TAACATTGCTGGCTGGATCCTGGGA-BHQ	"
**Influenza A (H3N2)**	H3hFor1	GGTACGGYTTCAGGCAT	"
	H3hRev1	TCAATCTGATGGAATTTCTCGTTG	"
	H3h-1144dProbe	FAM-CTGCTGCTTGTCCTCTTCCCT-BHQ	11
**Influenza B**	HAB-444fw	ACCCTACARAMTTGGAACYTCAGG	"
	HAB-524Rv	ACAGCCCAAGCCATTGTTG	"
	HAB-499Probe	VIC-ATCCGTTTCCATTGGTAA-MGBNFQ	"
	HAB-501bProbe	FAM-AAATCCGMTTTTAYTGGTAG-MGBNFQ	"
**Dengue 1**	DEN-1-forward	GACACCACACCCTTTGGACAA	12
	DEN-1-reverse	CACCTGGCTGTCACCTCCAT	"
	DEN-1-probe	FAM-AGAGGGTGTTTAAAGAGAAAGTTGACACGCG-TAMRA	"
**Dengue 2**	D2TaqC(b)-f	TTCATGGCCCTKGTGGC	13
	D2Cor05r	CCCCATCTYTTYARTATCCCTG	"
	D2TaqCor-FAM	FAM-TCCTTCGTTTCCTAACAATCC- TAMRA	12
**Dengue 3**	DEN-3-forward	GGGAAAACCGTCTATCAATA	"

	DEN-3-reverse	CGCCATAACCAATTTCATTGG	“
	DEN-3-probe	FAM-CACAGTTGGCGAAGAGATTCTCAAGAGGA-TAMRA	“
**Dengue 4**	DEN-4-forward	TGAAGAGATTCTCAACCGGAC	“
	DEN-4-reverse	AATCCCTGCTGTTGGTGGG	“
	DEN-4-probe	FAM-TCATCACGTTTTTGCGAGTCCTTTCCA-TAMRA	“
**Molluscum contagiosum**	MCVp43kF	GCTCACGTACGACTGCTTYGAC	[14]
	MCVp43kR	CGTGGAGCGCAGATTGC	“
	MCVp43kP	FAM-CGCTCATCTCGCAGAC-MGBNFQ	“
**Mumps**	Mumps For-HN-F	GGCCGTATGAACTCCTCTATGAGA	This study
	Mumps Rev-HN-R	AATGGCCAGGGATCAAGATAAAC	This study
	Mumps Probe-HN-TM	FAM-TGTCCTGGATACCTATATATTCATTCACTCGTCCTG-TAMRA	This study
**Rotavirus**	RotaA2016TM F1	ACCATCTACACATGACCCTC	[15]
	RotaA2016TM F2	ACCATCTTCACGTAACCCTC	“
	RotaA2016TM R	GGTCACATAACGCCC	“
	RotaA2016TM PRB	FAM-ATGAGCACAATAGTTAAAAGCTAACACTGTCAA-BHQ	“
**Zika**	Zika E For	AAGTTTGCATGCTCCAAGAAAAT	[16]
	Zika E Rev	CAGCATTATCCGGTACTCCAGAT	“
	Zika E Probe	FAM-ACCGGGAAGAGCATCCAGCCAGA-TAMRA	“

References

1 Huang, B., Pyke, A. T., McMahon, J. & Warrilow, D. Complete Coding Sequence of a Case of Chikungunya Virus Imported into Australia. *Genome Announc* **5**, doi:10.1128/genomeA.00310-17 (2017).

2 Lun, J. H. *et al.* Emerging recombinant noroviruses identified by clinical and waste water screening. *Emerg Microbes Infect* **7**, 50, doi:10.1038/s41426-018-0047-8 (2018).

3 Bhudevi, B. & Weinstock, D. Fluorogenic RT-PCR assay (TaqMan) for detection and classification of bovine viral diarrhea virus. *Vet Microbiol* **83**, 1-10 (2001).

4 Smith, G. in *PCR for Clinical Microbiology: An Australian and International Perspective* (ed Sloots TP Schuller M, James GS, Halliday CL, Cater, IWJ) Ch. 51, 313-315 (Springer, 2010).

5 van den Hurk, A. F., Hall-Mendelin, S., Pyke, A. T., Smith, G. A. & Mackenzie, J. S. Vector competence of Australian mosquitoes for chikungunya virus. *Vector Borne Zoonotic Dis* **10**, 489-495, doi:10.1089/vbz.2009.0106 (2010).

6 Mackay, I., Finger, M., Lyon, M. & Northill, J. *Hepatitis A MGB TaqMan*, <https://www.protocols.io/view/hepatitis-a-mgb-taqman-rk3d4yn?step=2> (2018).

7 Gerber, P. F., Xiao, C. T., Cao, D., Meng, X. J. & Opriessnig, T. Comparison of real-time reverse transcriptase PCR assays for detection of swine hepatitis E virus in fecal samples. *J Clin Microbiol* **52**, 1045-1051, doi:10.1128/JCM.03118-13 (2014).

8 Kageyama, T. *et al.* Broadly reactive and highly sensitive assay for Norwalk-like viruses based on real-time quantitative reverse transcription-PCR. *J Clin Microbiol* **41**, 1548-1557 (2003).

9 Smith, I. L., Northill, J. A., Harrower, B. J. & Smith, G. A. Detection of Australian bat lyssavirus using a fluorogenic probe. *J Clin Virol* **25**, 285-291 (2002).

10 Huang, B. *et al.* Diagnosis and typing of influenza using fluorescent barcoded probes. *Sci Rep* **7**, 18092, doi:10.1038/s41598-017-18333-7 (2017).

11 WHO. CDC protocol of real-time RT-PCR for influenza A (H1N1). (2009).

12 Callahan, J. D. *et al.* Development and evaluation of serotype- and group-specific fluorogenic reverse transcriptase PCR (TaqMan) assays for dengue virus. *J Clin Microbiol* **39**, 4119-4124, doi:10.1128/JCM.39.11.4119-4124.2001 (2001).

13 Mackay, I., Northill, J. & Pyke, A. *Dengue virus type 2 (DENV-2) capsid-Thai TaqMan assay*, <https://www.protocols.io/view/dengue-virus-type-2-denv-2-capsid-thai-taqman-assa-q4ydyxw> (2018).

14 Northill, J., Simmons, R. & Mackay, I. *Molluscum contagiosum virus real-time PCR*, <https://www.protocols.io/view/molluscum-contagiosum-virus-real-time-pcr-kmtcu6n> (2017).

15 Ye, S. *et al.* Evidence of false-positive results in a commercially available rotavirus assay in the vaccine era, Australia, 2011 to 2012. *Euro Surveill* **18** (2013).

16 Pyke, A. T. *et al.* Imported zika virus infection from the cook islands into australia, 2014. *PLoS Curr* **6**, doi:10.1371/currents.outbreaks.4635a54dbffba2156fb2fd76dc49f65e (2014).
